# Functional Inhibition of Host Histone Deacetylases (HDACs) Enhances *in vitro* and *in vivo* Anti-mycobacterial Activity in Human Macrophages and in Zebrafish

**DOI:** 10.3389/fimmu.2020.00036

**Published:** 2020-02-03

**Authors:** Jôsimar D. Moreira, Bjørn E. V. Koch, Suzanne van Veen, Kimberley V. Walburg, Frank Vrieling, Tânia Mara Pinto Dabés Guimarães, Annemarie H. Meijer, Herman P. Spaink, Tom H. M. Ottenhoff, Mariëlle C. Haks, Matthias T. Heemskerk

**Affiliations:** ^1^Department of Infectious Diseases, Leiden University Medical Center, Leiden, Netherlands; ^2^Department of Clinical and Toxicological Analysis, Faculty of Pharmacy, Federal University of Minas Gerais, Belo Horizonte, Brazil; ^3^Institute of Biology Leiden, Leiden University, Leiden, Netherlands

**Keywords:** tuberculosis, host-directed therapy, epigenetic regulation, histone deacetylases (HDAC), human macrophages

## Abstract

The rapid and persistent increase of drug-resistant *Mycobacterium tuberculosis* (*Mtb*) infections poses increasing global problems in combatting tuberculosis (TB), prompting for the development of alternative strategies including host-directed therapy (HDT). Since *Mtb* is an intracellular pathogen with a remarkable ability to manipulate host intracellular signaling pathways to escape from host defense, pharmacological reprogramming of the immune system represents a novel, potentially powerful therapeutic strategy that should be effective also against drug-resistant *Mtb*. Here, we found that host-pathogen interactions in *Mtb*-infected primary human macrophages affected host epigenetic features by modifying histone deacetylase (HDAC) transcriptomic levels. In addition, broad spectrum inhibition of HDACs enhanced the antimicrobial response of both pro-inflammatory macrophages (Mϕ1) and anti-inflammatory macrophages (Mϕ2), while selective inhibition of class IIa HDACs mainly decreased bacterial outgrowth in Mϕ2. Moreover, chemical inhibition of HDAC activity during differentiation polarized macrophages into a more bactericidal phenotype with a concomitant decrease in the secretion levels of inflammatory cytokines. Importantly, *in vivo* chemical inhibition of HDAC activity in *Mycobacterium marinum*-infected zebrafish embryos, a well-characterized animal model for tuberculosis, significantly reduced mycobacterial burden, validating our *in vitro* findings in primary human macrophages. Collectively, these data identify HDACs as druggable host targets for HDT against intracellular *Mtb*.

## Introduction

Tuberculosis (TB) is a health threat of global dimensions, and is caused by the highly successful human pathogen *Mycobacterium tuberculosis* (*Mtb*). Remarkably, *Mtb* is capable of establishing intracellular infection even in the presence of strong innate and adaptive host immunity. One fourth of the global human population is estimated to be latently infected with *Mtb*. These individuals have a 5–10% lifetime risk of developing TB reactivation disease, resulting in 10 million people falling ill with TB and over 1.5 million deaths each year (1). In HIV-infected or otherwise immunocompromised patients the risk of TB reactivation is significantly increased.

Current interventions (antibiotics, BCG vaccination) fail to reduce TB incidence sufficiently. Together with the rising frequency of multi-, extensively-, and even totally drug-resistant (MDR/XDR/TDR) *Mtb* strains, and the fact that many druggable targets in pathogens are already inhibited by current antibiotics (2), it is crucial to develop new and much more effective strategies that act by mechanisms different from those already targeted by current interventions. Since *Mtb* has a remarkable ability to manipulate intracellular signaling pathways which promote its escape from host defense in human cells, host-directed therapies (HDT) would represent a therapeutic strategy that would be effective also against currently untreatable strains since these compounds act on host and not on pathogen molecules.

TB most commonly presents as a pulmonary disease following inhalation of *Mtb*-containing droplets in the lung. Modulation of host signaling pathways by *Mtb* in infected alveolar macrophages arrests phagosome maturation to create a niche for its intracellular survival (3, 4). In addition, activation of alveolar macrophages results in transcriptional changes that regulate innate and adaptive immune responses such as production of chemokines and pro- and anti-inflammatory cytokines (5). Epigenetic regulators play a crucial role in regulating the transcriptional response to microorganisms by chromatin remodeling (6, 7). Acetylation of histone proteins is one of the main mechanisms to control DNA accessibility and thereby gene expression (8). Histone acetyltransferases (HATs) acetylate lysine residues in histone tails resulting in a more relaxed chromatin structure which is associated with transcriptional activation. In contrast, histone-deacetylases (HDACs) counteract the activity of HATs by removing acetyl groups from highly conserved lysine residues resulting in more condensed chromatin structure which is associated with transcriptional repression by limiting the accessibility to the transcriptional machinery. HDACs are divided into four classes: Class I (HDAC1, 2, 3, and 8), class II (class IIa HDAC4, 5, 7, and 9; class IIb HDAC6 and 10), class III (SIRT1-7), and class IV (HDAC11) based on their function, co-factor dependency and structural homology to yeast HDACs (9). Class I, II, and IV enzymes belong to the family of “classical” HDACs and have a zinc-dependent active site, whereas class III proteins are NAD^+^-dependent and considered a family of “non-classical” HDACs.

HDACs are important players in the differentiation of macrophages and their role in immunity. HDAC3 has been shown to be vital in the development of anti-inflammatory macrophages (Mϕ2) by repressing alternative macrophage activation (10) whereas pro-inflammatory macrophages (Mϕ1) are impacted by several HDACs, such as HDAC4, 5, 6, and 7, which strongly regulate the expression of pro-inflammatory genes upon stimulation with e.g., lipopolysaccharide (LPS) (11–13). Granuloma formation in the lungs of TB infected individuals is driven by macrophages and the resulting outcome of infection, i.e., bacterial control or bacterial dissemination, relies on macrophage type, and polarization (14, 15). It is therefore not surprising that several pathogens, including *Mtb* have been implicated in evading the immune system by modulating histone acetylation via altering HDAC expression levels (11, 16–19).

In the present study, we investigated the expression kinetics of different classes of HDAC transcripts in response to *Mtb* infection in primary human macrophages and found expression levels of a diverse set of HDAC genes to be affected by *Mtb*. We next investigated the impact of HDAC inhibition on infection in human macrophages *in vitro*. A pan-HDAC inhibitor as well as several selective class IIa inhibitors significantly reduced outgrowth of intracellular *Mtb* in macrophages. Importantly, these results were validated in an *in vivo* model of tuberculosis, the *Mycobacterium marinum* (*Mmar*) zebrafish embryo infection model (20–22). Collectively these results establish the potential of HDAC inhibitors as novel host-directed therapeutics for TB.

## Materials and Methods

### Reagents

H-89 dihydrochloride (PKA/PKB/AKT1 kinase inhibitor), 3-aminobenzoic acid ethyl ester (tricaine), and rifampicin were purchased from Sigma-Aldrich, Zwijndrecht, The Netherlands. H-89 analog 97i was synthesized by the Leiden Academic Center for Drug Research, Division of Medicinal Chemistry, Leiden University, Leiden, The Netherlands. Pan-HDAC inhibitor Trichostatin A (TSA) and class IIa HDAC inhibitors TMP195 and TMP269 were purchased from Selleckchem, Munich, Germany. Hygromycin B was acquired from Life Technologies-Invitrogen, Bleiswijk, The Netherlands. Recombinant human IFN-γ protein was acquired from R&D Systems, Wiesbaden, Germany.

### Cell Culture

Peripheral blood mononuclear cells (PBMCs) were isolated from buffy coats obtained from healthy donors after written informed consent (Sanquin Blood Bank, Amsterdam, The Netherlands). Monocytes were isolated through density gradient centrifugation over Ficoll-Paque followed by CD14 MACS sorting (Miltenyi Biotec, Bergisch Gladsbach, Germany) and differentiated for 6 days into pro-inflammatory (Mϕ1) or anti-inflammatory (Mϕ2) macrophages with 5 ng/ml of granulocyte-macrophage colony-stimulating factor (GM-CSF; Life Technologies-Invitrogen) or 50 ng/ml macrophage colony-stimulating factor (M-CSF; R&D Systems, Abingdon, UK), respectively, as previously reported (23). Cells were cultured at 37°C/5% CO_2_ in Gibco Roswell Park Memorial Institute (RPMI) 1640 medium (Life Technologies-Invitrogen) supplemented with 10% FBS and 2 mM L-alanyl-L-glutamine (GlutaMAX) (PAA, Linz, Austria), 100 U/ml penicillin, and 100 μg/ml streptomycin (Life Technologies-Invitrogen). Macrophage differentiation and activation status was determined by quantifying IL-12p40 and IL-10 secretion (for Mϕ1 and Mϕ2, respectively) by ELISA following stimulation of cells in the presence or absence of 100 ng/ml lipopolysaccharide (LPS) for 24 h (InvivoGen, San Diego, United States).

### *Mtb* Infection of Macrophages

*Mtb* [DsRed-expressing H37Rv (24)] was cultured in Difco Middlebrook 7H9 broth (Becton Dickinson, Breda, The Netherlands) supplemented with 10% ADC (Becton Dickinson) and 0.05% Tween 80 (Sigma-Aldrich). One day before infection, *Mtb* cultures were diluted to a density corresponding with early log-phase growth (OD_600_ of 0.25). The following day, bacterial suspensions (or 7H9 for mock infections) were diluted in cell culture medium without antibiotics to reach a multiplicity of infection (MOI) of 10. MOI of the inoculum was verified by a standard colony-forming unit (CFU) assay. Cells seeded in 96-well flat-bottom plates at a density of 30,000 cells/well in appropriate cell culture medium without antibiotics 1 day prior to infection, were inoculated with 100 μl of the bacterial suspension, centrifuged for 3 min at 800 rpm, and incubated at 37°C/5% CO_2_ for 60 min. Bacteria were then washed away with cell culture medium containing 30 μg/ml gentamicin sulfate (Lonza BioWhittaker, Basel, Switzerland), incubated for 10 min at 37°C/5% CO_2_, followed by replacement with medium containing 5 μg/ml gentamicin sulfate and, if indicated, chemical compounds until readout by flow cytometry, Luminex, or CFU.

### Chemical Compound Treatment

During differentiation, monocytes were treated for 6 days with 300 nM TMP195, 300 nM TMP269, 30 nM TSA, or DMSO at equal v/v (25). The 300 nM concentration used for TMP195 and TMP269 was based on results reported by Guerriero et al. (26) in a similar monocyte differentiation model and was not toxic. TSA was used at a concentration of 30 nM for 6 days because higher concentrations showed toxicity. Alternatively, *Mtb*-infected Mϕ1 and Mϕ2 were treated for 48 h with 10 μM TMP195, TMP269, H-89 and 97i, 100 nM TSA, or DMSO at equal v/v in medium containing 5 μg/ml gentamicin sulfate. Prior to these experiments, we had performed pilot experiments to exclude cellular toxicity on primary human macrophages. We found no toxicity for both TMP195 and TMP269 at 10 μM concentrations, which constitutes a standard concentration in initial drug screening, confirming results from Lobera et al. (25). Trichostatin A, however, was found to be highly toxic at 10 μM and was therefore evaluated at lower concentrations. A concentration of 0.1 μM was found to be non-toxic in our primary human macrophage model, agreeing well with previously published results (27).

### Zebrafish Handling, Compound Treatment, and *Mycobacterium marinum* Infection

Zebrafish were handled in compliance with animal welfare regulations and maintained according to standard protocols (http://zfin.org). Fertilized embryos were maintained at 28°C and kept in egg water [60 μg/ml Instant Ocean Sea Salt (Sera, Heinsberg Germany)]. Zebrafish embryos starting the 20 somite stage were exposed for the following 24 h to 10 μM TMP195, 30 nM TSA, or DMSO at equal v/v in egg water at 28°C. *Mycobacterium marinum* (*Mmar*) M-strain carrying a plasmid encoding the Wasabi fluorescent protein (28) was cultured in 7H9 medium with 10% BBL ADC enrichment medium (Becton Dickinson, Franklin Lakes, United States) and 50 μg/ml Hygromycin at 28°C, to an optical density OD_600_ of ~1. For the duration of bacterial injections, zebrafish larvae were kept under anesthesia in egg water containing 0.02% buffered 3-aminobenzoic acid ethyl ester (tricaine) and infections were performed by microinjection of 250–300 CFU into the Duct of Cuvier at ~43 h post fertilization (hpf), 24 h post compound treatment, as previously described (29). At 3 days post infection (dpi), the infection was quantified by fluorescent pixel determination (30). Infected embryos were anesthetized using 0.02% tricaine in egg water and imaged using a Leica MZ16FA Fluorescence Stereo Microscope (Leica Microsystems, Wetzlar, Germany) equipped with a DFC420C color camera (Leica Microsystems, Wetzlar, Germany).

### Colony-Forming Unit (CFU) Assay

CFU spot assays have been described elsewhere (31). Briefly, cells were lysed in H_2_O containing 0.05% SDS. Cell lysates were serially diluted in multiple steps of 5-fold dilutions in 7H9 broth and 10 μl droplets were spotted onto square Middlebrook 7H10 agar plates and incubated for 12–14 days at 37°C. Bacterial colonies were enumerated using a microscope with a 2.5x magnification to enhance early detection of bacterial growth.

### *Mtb* Growth Assay

A volume of 100 μl of *Mtb* culture (OD_600_ of 0.2) in 7H9 broth was plated in a flat-bottom 96-well plate containing 100 μl of 7H9 broth with TMP195, TMP269, TSA, or Rifampicin as a positive control or DMSO at equal v/v at indicated concentrations. Growth was evaluated at 37°C for 13 days and absorbance was measured by optical density at 550 nm on a Mithras LB 940 plate reader (Berthold Technologies, Bad Wildbad, Germany).

### Flow Cytometry

Single cell suspensions were incubated for 5 min with 5% human serum (Sanquin Blood Bank) in PBS to block non-specific Fc-receptor binding, washed in PBS/0.1% BSA (Merck, Darmstadt, Germany) and stained with monoclonal antibodies against cell surface markers CD11b-PE, CD1a-BV605, CD80-FITC, CD86-AF700 (all BD BioSciences, Vianen, The Netherlands), CD14-FITC, and CD163-AF647 (BioLegend, San Diego, CA, USA) for 30 min at 4°C. Cells were washed twice in PBS/0.1% BSA and acquisition was performed using a BD FACSLyric™ Flow Cytometer (BD Biosciences). Data was analyzed using FlowJo v10 software.

### Cell Viability Assay

Cells seeded at a density of 30,000 cells/well in 96-well flat-bottom plates were stained in 50 μl cell culture medium without phenol red containing propidium iodide (PI) (1:500, Sigma-Aldrich) and Hoechst (1:100, Sigma-Aldrich). After incubation for 5 min at room temperature (RT), 3 images per well were recorded using a Leica AF6000 LC fluorescence microscope combined with a 20x dry objective. Cell viability was calculated by quantifying the number of dead cells (PI positive) vs. total cell numbers (Hoechst positive) using ImageJ software.

### Microscopy

Bright field image acquisition was performed using an Olympus IX51 Inverted Microscope combined with Olympus cellSens software.

### Cytokine and Chemokine Multiplex Beads Assay

Culture supernatants were collected 24 h post-infection and filter-sterilized by centrifugation in 96-well filter plates containing a 0.2 μm PVDF membrane (Corning, Amsterdam, The Netherlands). Forty-one analytes were quantified using the Milliplex Human Cytokine/chemokine magnetic bead premixed 41-plex kit (Millipore Billerica, MA, USA) according to the manufacturer's instructions. Analyses were performed on a Bio-Plex 100 with Bio-Plex Manager™ software v6.1 (Biorad, Veenendaal, The Netherlands).

The following analytes were measured: sCD40L, EGF, FGF-2, Flt3 ligand, Fractalkine (CX3CL1), G-CSF, GM-CSF, GRO (CXCL1), IFN-γ, IFN-α2, IL-1α, IL-1β, IL-1ra, IL-2, IL-3, IL-4, IL-5, IL-6, IL-7, IL-8 (CXCL8), IL-9, IL-10, IL-12p40, IL-12p70, IL-13, IL-15, IL-17a, IP-10 (CXCL10), MCP-1 (CCL2), MCP-3 (CCL7), MDC (CCL22), MIP-1α (CCL3), MIP-1β (CCL4), PDGF-AB/BB, RANTES (CCL5), TGF-α, TNF-α, TNF-β, VEGF, Eotaxin (CCL11), and PDGF-AA.

### Phagocytosis Assay

Cells were pulsed with Fluoresbrite® YG Carboxylate Microspheres P beads (Polysciences, Warrington, PA, USA) in a ratio of 10 beads to 1 cell for 90 min at 37°C/5% CO_2_. Cells were subsequently washed with PBS and harvested by adding Trypsin-EDTA 0.5% (ThermoFisher Scientific, Waltham, MA, USA). Cells were centrifuged and resuspended in 100 μl Trypan Blue (1:1) in PBS/0.1% BSA (Merck) to quench fluorescence of extracellular beads. Internalized beads were quantified by flow cytometry on a BD FACSLyric™. Data analysis was performed using FlowJo v10 software.

### Total RNA Isolation and cDNA Synthesis

Total RNA isolation was performed using TRIzol Reagent (Life Technologies-Invitrogen) according to the manufacturer's instructions and RNA yield was quantified using a DeNovix DS-11 Spectrophotometer (ThermoFisher Scientific). Total RNA (0.5 μg) was reverse transcribed using SuperScript IV Reverse Transcriptase (Life Technologies-Invitrogen). Briefly, RNA samples were first incubated at 65°C for 5 min in the presence of 0.5 mM dNTPs and 2.5 μM oligo(dT)_20_ (Life Technologies-Invitrogen). Subsequently, cDNA synthesis was initiated by adding a master mix containing 1x first strand buffer, 5 mM DTT, 40 U RNaseOUT (ThermoFisher Scientific), and 200 U SuperScript IV and incubating at 50–55°C for 10 min followed by inactivation of the reverse transcriptase at 80°C for 10 min.

### TaqMan qPCR

Multiplex quantitative polymerase chain reaction (qPCR) was carried out using a QuantStudio 6 Flex Real-Time PCR System (ThermoFisher Scientific). qPCR reactions were performed in a final volume of 25 μl containing 1x TaqMan Universal PCR Master Mix, No AmpErase UNG, 1x HDAC(1-11)-FAM TaqMan primers (ThermoFisher Scientific), 0.5x GAPDH-VIC TaqMan primers (ThermoFisher Scientific), and 20 ng cDNA. Thermal cycling conditions were 1 cycle of 2 min/50°C and 10 min/95°C, followed by 60 cycles of 15s/95°C and 1 min/60°C. The threshold cycle (Ct) values of HDAC transcripts were normalized to GAPDH by the 2^−ΔΔ*CT*^ algorithm method (32). Relative expression levels were calculated by applying the formula ((2^−Δ*CT*(*Target gene*)^)/(2^−Δ*CT*(*GAPDH*)^)). The following TaqMan® Gene Expression Assays were used: GAPDH-VIC (Hs02758991_g1), HDAC1-FAM (Hs00606262_g1), HDAC2-FAM (Hs00231032_m1), HDAC3-FAM (Hs00187320_m1), HDAC4-FAM (Hs01041638_m1), HDAC5-FAM (Hs00608351_m1), HDAC6-FAM (Hs00997427_m1), HDAC7-FAM (Hs00248789_m1), HDAC8-FAM (Hs00954353_g1), HDAC9-FAM (Hs01081558_m1), HDAC10-FAM (Hs00368899_m1), HDAC11-FAM (Hs00978038_m1), TNF-FAM (Hs00174128_m1), IL6-FAM (Hs00174131_m1), CSF3-FAM (Hs00738432_g1), IFNG-FAM (Hs00989291_m1), CCL2-FAM (Hs00234140_m1), CCL3-FAM (Hs00234142_m1), CCL4-FAM (Hs99999148_m1), and CXCL8-FAM (Hs00174103_m1). TNF, IL6, CSF3, and IFNG could not be detected within a cycle threshold (Ct) of 45.

### Data Analysis

Normal distribution of data sets was evaluated using the Shapiro-Wilk normality test. Paired sample *t*-test analysis was employed when comparing two experimental conditions. One-way ANOVA and repeated measure (RM) one-way ANOVA with Dunnett's multiple test correction were applied when assessing differences between 3 or more groups of unpaired and paired samples, respectively. Kruskal-Wallis test followed by Dunnett's multiple test correction was used when comparing non-parametric data sets of 3 or more groups. All analyses were performed using GraphPad Prism 8.

For multilevel partial least squares-discriminant analysis (PLS-DA) (33), the R package mixOmics (version 6.3.2) was used (34). Model validity was assessed by determining model quality characteristics for explained variance (R^2^X, R^2^Y) and predictive ability (${\text{Q}}_{\text{cum}}^{2}$) after leave-one-out cross validation (LOOCV). Variable Importance in Projection (VIP) scores of the first x-variate, representing the contribution of each variable to the model, were extracted from each PLS-DA analysis and values ≥1 were considered relevant. Only analytes that changed in at least 3 out of 4 donors with a minimal median log_2_ fold change (FC) of 0.5 were included in the analyses. The associations of analytes with treatment response are reflected by Kendall correlation coefficients. For calculation of the Kendall rank correlation coefficient tau-b, the R package Kendall (version 2.2) was used (35).

Fluorescent images of infected zebrafish embryos in Tiff file format were processed and quantified using the Fiji distribution of ImageJ (36). For the processing steps, involving thresholding and quantification of positive pixels, an ImageJ macro was developed: run(“8-bit”); setAutoThreshold(“Triangle dark”); setThreshold(4, 255); run(“Convert to Mask”); run(“Measure”); close().

## Results

### Regulation of HDAC Transcriptomic Profiles in Response to *Mtb* Infection

**Figure 1:**
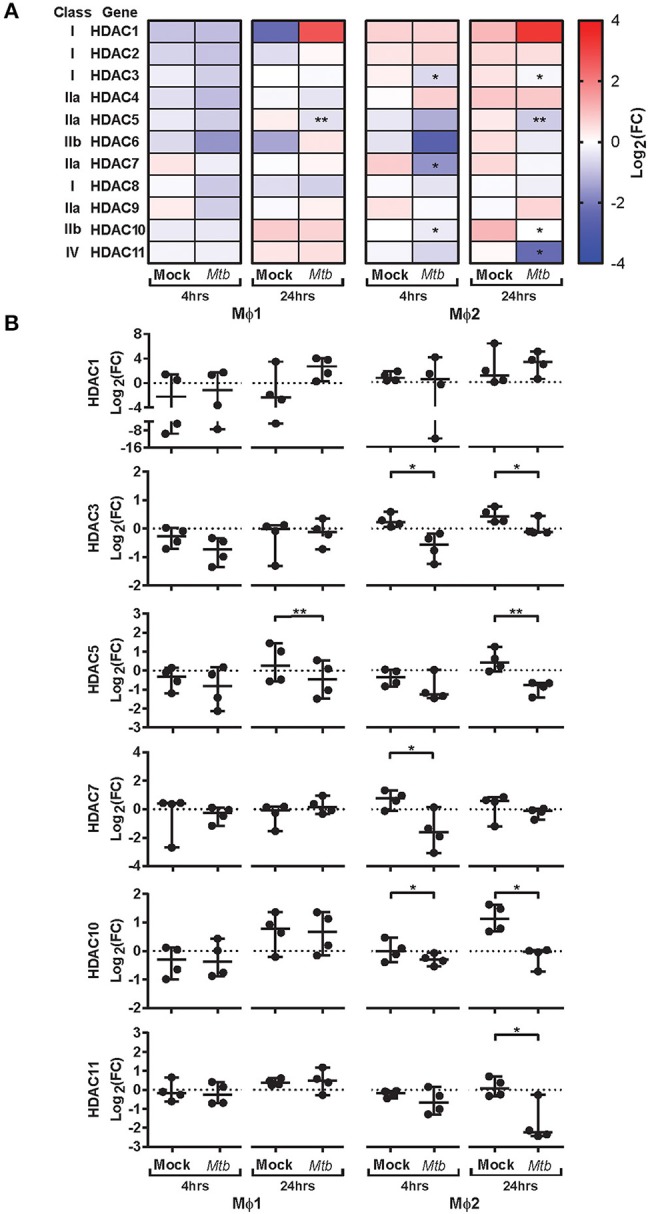
Expression kinetics of HDAC transcripts in primary human macrophages following *Mtb* infection. Mϕ1 and Mϕ2 derived from 4 different donors were mock infected or infected with *Mtb* for 1 h at MOI 10. Transcript levels of HDAC1-11 were determined in triplicate using qRT-PCR before (0 h baseline) and 4 and 24 h post-infection. Data was normalized to GAPDH (ΔCt) and mean expression levels of triplicate samples were calculated for each donor. **(A)** Heatmap displaying median log_2_ fold changes (FC) of HDAC1-11 expression levels (pooled data of 4 donors) in response to *Mtb* infection compared to their respective baseline controls. **(B)** Dot plots displaying log_2_ FC expression levels of HDAC1, 3, 5, 7, 10, and 11 in response to *Mtb* infection compared to their respective baseline controls, calculated using the 2^−ΔΔ*CT*^ formula. Each dot represents a single donor. Horizontal lines indicate median log_2_ FC values of all 4 donors and whiskers represent 95% confidence intervals. Statistically significant differences compared to uninfected controls were tested using a paired sample *t*-test (*p < 0.05, **p < 0.01).

To explore whether intracellular survival of *Mtb* is controlled by host epigenetic features, we investigated whether *Mtb*-H37Rv (*Mtb*) infection could impact histone acetylation in primary human pro-inflammatory (Mϕ1) and anti-inflammatory (Mϕ2) macrophages (the main target cell of *Mtb*), representing opposing ends of the macrophage differentiation spectrum. Expression kinetics of all 11 canonical HDAC transcripts were determined in triplicate by qRT-PCR before (baseline) and 4 and 24 h following infection with *Mtb* (Figure 1). Differential regulation of HDAC transcript levels upon *Mtb* infection was more pronounced in Mϕ2 than Mϕ1. HDAC1 was substantially upregulated 24 h post-infection in both Mϕ1 and Mϕ2. In contrast, expression levels of HDAC3, 5, 7, 10, and 11 were significantly repressed in Mϕ2 whereas in Mϕ1 this was only observed for HDAC5. Interestingly, expression levels of 4 out of 5 HDACs that were significantly suppressed in Mϕ2 following infection with *Mtb*, exhibited significantly higher transcript levels in Mϕ2 compared to Mϕ1 at baseline (Figure S1A). Since HDACs are considered molecular switches regulating a plethora of processes including balancing pro-vs. anti-inflammatory responses (Table 1), distinct baseline expression levels of HDAC family members might explain differences in inflammatory cytokine profiles between activated Mϕ1 and Mϕ2 (23). Using a published RNA-sequencing dataset of *Mtb-*infected Mϕ2, we were able to independently validate our findings [markedly enhanced expression levels of HDAC1 and significantly reduced transcript levels of HDAC3, 5, 10, and 11 upon infection with *Mtb*-H37Rv, heat-killed *Mtb*-H37Rv and Bacillus Calmette-Guérin (BCG)] for anti-inflammatory macrophages (Figure S1B) (64). Interestingly, lower expression levels of HDAC3, 5, 10, and 11 were not seen at early timepoints after infection with *Mtb*-GC1237, a virulent Beijing strain, implying that this could be advantageous to the pathogen. This suggests that lowering these specific HDAC expression levels might be beneficial to the host. Together, these data suggest that host-pathogen interactions in *Mtb*-infected macrophages affect host epigenetic features by modifying histone acetylation through regulating HDAC expression levels. Therefore, targeting HDACs with small molecules could potentially regulate outgrowth of intracellular infections with *Mtb*.

**Table 1:** Regulation of cytokine/chemokine expression by HDACs in phagocytes.

**Class**	**HDAC enzyme**	**Analyte**	**Immunostimulatory agent**	**Method of interference**	**Cell type**	**References**
I	HDAC1	IL-1β	LPS + IFN-γ	Genetic (siRNA)	RAW 264.7	(37)
		IL-12p40	*Mtb*	Genetic (siRNA)	THP-1	(18)
		IL-8	LPS	Genetic (siRNA)	GM-CSF differentiated human Mϕ	(38)
		IL-1β,IL-10, IL12p40, TNF-α	LPS + IFN-γ	Chemical (MS-275)	RAW 264.7	(39)
		IL-1β, IL-10	LPS	Chemical (MS-275)	RAW 264.7	(40)
		IL-6, IL-10, IL-12, TNF-α	poly(I:C)	Chemical (MS-275)	IL-4 + GM-CSF differentiated human DC	(41)
I	HDAC2	IL-1β, IL-1ra	LPS	Chemical (Ky-2)	THP-1	(42)
		MIP-2α, TNF-α	LPS	Genetic (overexpression)	RAW 264.7, PMϕ	(43)
				Chemical (Theophylline)		
		IL-12p70, TNF-α	LPS	Genetic (shRNA)	RAW 264.7, BMMϕ	(44)
		IL-6	LPS	Genetic (siRNA)	IL-4 + GM-CSF differentiated BMDC	(45)
I	HDAC3	IL-1β, IL-6, IL-10, IL-12p40	LPS + IFNγ	Genetic (siRNA)	RAW 264.7	(37)
				Chemical (RGFP966)		
		IL-1β, IL-8	LPS	Genetic (siRNA)	GM-CSF differentiated human Mϕ	(38)
		IL-6	LPS	Chemical (Scriptaid/RGFP966)	LPS differentiated BMMϕ	(46)
		IL-6, IFN-β	LPS	Genetic (KO mice)	BMMϕ	(47)
		TNF-α	LPS	Genetic (siRNA/overexpression)	U937	(48)
IIa	HDAC4	IL-6, TNF-α	LPS	Genetic (siRNA)	BV2	(49)
IIa	HDAC5	IL-6, IL-10, MCP-1, TNF-α	LPS	Genetic (siRNA/overexpression)	RAW 264.7	(50)
		IL-1β, IL-6, TNF-α	*Mycoplasma pneumoniae*	Genetic (siRNA/overexpression)	THP-1	(51)
IIb	HDAC6	IL-10, IL-12p40, IFN-γ	*Mtb*	Genetic (siRNA/overexpression)	THP-1	(52)
		IL-1β	LPS + ATP	Genetic (shRNA)	BMMϕ	(53)
		IL-1β, IL-6, TNF-α	Unstimulated	Genetic (overexpression)	RAW 264.7	(54)
				Chemical (Tubastatin A)		
		IL-6, TNF-α	LPS	Chemical (Tubastatin A)	RAW 264.7, M-CSF differentiated BMMϕ	(13)
		IL-10	LPS	Genetic (shRNA)	RAW 264.7, PMϕ	(55)
					IL-4 + GM-CSF differentiated BMDC	
		IL-10	LPS	Genetic (overexpression)	RAW 264.7, THP-1	(56)
					IL-4 + GM-CSF differentiated human DC	
		IL-6, TNF-α	LPS	Chemical (Tubastatin A)	THP-1	(57)
IIa	HDAC7	IL-6, IL-1β, IL-10, IL12p, TNF-α	LPS	Genetic (overexpression)	RAW 264.7	(12)
				Chemical (Compound 6a)		
I	HDAC8	IL-6^*^, IL-1β, TNF-α^*^	LPS	Chemical (WK2-16)	THP-1	(58)
IIa	HDAC9	IL-1β, IL-6, TNF-α	LPS	Genetic (siRNA)	RAW 264.7	(59)
IIb	HDAC10	Unknown				
IV	HDAC11	IL-10, IL-1β, IL-10, IL12p, IFN-γ	*Mtb*	Genetic (siRNA/overexpression)	THP-1	(52)
		IL-10	LPS	Genetic (shRNA)	RAW 264.7	(55)
		IL-10, IL-12p70	LPS	Genetic (overexpression)	RAW 264.7, THP-1	(56)
					IL-4 + GM-CSF differentiated human DC	
		IL-10, IL-12p70	*Leishmania donovani*	Genetic (overexpression)	RAW 264.7	(60)
		IL-10, IL-12p70	Unstimulated	Genetic (siRNA/overexpression)	THP-1, NOMO-1	(61)
		IL-10	LPS	Genetic (siRNA)	RAW 264.7	(62)
		IL-10, IL-12p70	LPS	Genetic (siRNA)	PMϕ	(63)

*Positive regulation of cytokines/chemokines is shown in red, negative regulation is indicated in blue*.

* *Findings have been validated in vivo in mice. PMϕ, peritoneal elicited macrophages; BMMϕ, bone marrow-derived macrophages; BMDC, bone marrow-derived dendritic cells*.

### Chemical Inhibition of HDACs Markedly Reduces Intracellular Survival of *Mtb*

**Figure 2:**
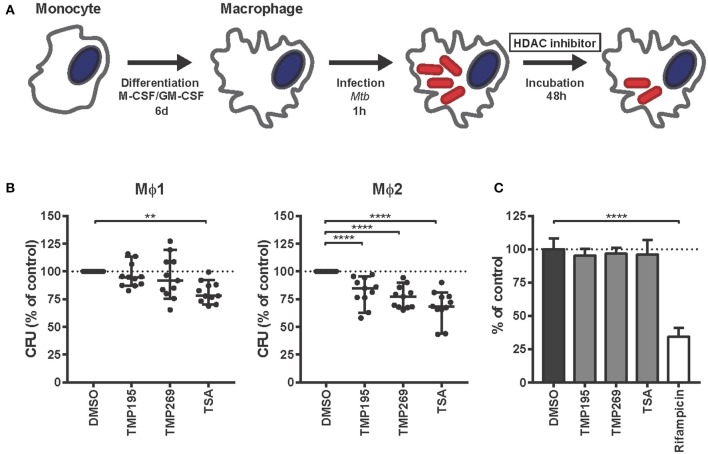
HDAC inhibitors decrease *Mtb* survival in human macrophages in a host-directed manner. **(A)** Schematic outline of the experimental setup used in **(B)**. **(B)** Mϕ1 and Mϕ2 macrophages derived from 11 different donors were infected with *Mtb* for 1 h at MOI 10 and treated with TMP195 (10 μM), TMP269 (10 μM), TSA (100 nM), or DMSO at equal v/v for 48 h post-infection. Dots represent the mean of 3 CFU assay replicates of a single donor expressed as a percentage of the DMSO control. Horizontal lines indicate median CFU values of all 11 donors and whiskers represent 95% confidence intervals. Statistically significant differences compared to DMSO were tested using a RM one-way ANOVA (**p < 0.01, ****p < 0.0001). **(C)** Thirteen days treatment of a *Mtb* broth culture in the presence of 10 μM TMP195, TMP269, or TSA. Rifampicin (20 μg/ml) was used as a positive control. Bars depict the mean bacterial density at 550 nm ± standard deviation of six replicates from a representative experiment out of two independent experiments. The bacterial load is expressed as a percentage of the DMSO control value. Statistically significant difference compared to DMSO was tested using a one-way ANOVA with Dunnett's multiple test correction (****p < 0.0001).

To investigate whether histone acetylation/deacetylation controls *Mtb* infection, *Mtb*-infected Mϕ1 and Mϕ2 were treated with selective class IIa HDAC inhibitors [TMP195 and TMP269 (25)] or the pan-HDAC inhibitor Trichostatin A (TSA) (Figure 2A). Given the nature and mechanisms of action of the HDAC targets (which enzymatically control epigenetic state), we hypothesized that a higher end dose of inhibitors was needed to be able to measure a phenotype in *Mtb*-infected cells (65). Pan-HDAC inhibition by TSA significantly reduced bacterial load in both Mϕ1 and Mϕ2 while selective inhibition of class IIa HDACs by TMP195 and TMP269 decreased intracellular *Mtb* outgrowth predominantly in Mϕ2 (Figure 2B). None of the compounds directly affected bacterial growth in liquid bacterial cultures while a suboptimal dose of the classical *Mtb* antibiotic rifampicin significantly inhibited *Mtb* (Figure 2C), confirming that HDAC inhibitors solely act via host-directed mechanisms and lack direct antimicrobial activity. Collectively, these data identify HDAC enzymes as a novel and important class of proteins in host regulatory networks that control intracellular bacterial survival. Furthermore, targeting HDACs with small molecules to regulate downstream inflammatory pathways could potentially be a novel host-directed therapeutic option for *Mtb* infections.

### HDAC Inhibition During Differentiation Polarizes Macrophages Into a More Bactericidal Phenotype

**Figure 3:**
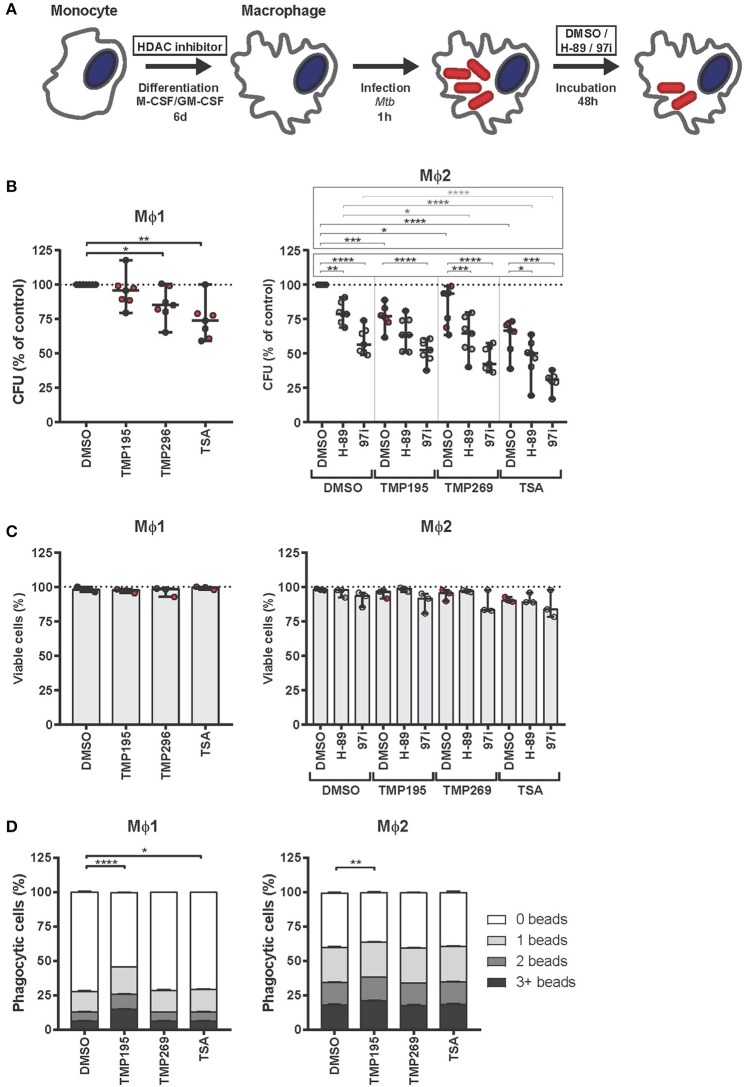
Macrophages exposed during differentiation to low concentrations of HDAC inhibitors are more bactericidal. **(A)** Outline of the experimental setup used in **(B–D)**. **(B)** Monocytes derived from 7 different donors were differentiated toward Mϕ1 and Mϕ2 while being exposed to TMP195 (300 nM), TMP269 (300 nM), TSA (30 nM), or DMSO at equal v/v for 6 days. Differentiated Mϕ1 and Mϕ2 were subsequently infected with *Mtb* for 1 h at MOI 10 and incubated for 48 h post-infection until readout. Dots represent the mean of 3 CFU assay replicates of a single donor expressed as a percentage of the DMSO control. Horizontal lines indicate median CFU values of all 7 donors and whiskers represent 95% confidence intervals. Statistically significant differences compared to DMSO were tested using a RM one-way ANOVA (*p < 0.05; **p < 0.01; ***p < 0.001; ****p < 0.0001). **(C)** Cell viability measurement of *Mtb*-infected Mϕ1 and Mϕ2 (experimental setup as in **B**). Dots represent the mean of 3 cell viability assay replicates of a single donor expressed as a percentage of the DMSO control. Bars indicate median values of all 3 donors and whiskers represent 95% confidence intervals. Statistically significant differences compared to DMSO were tested using a RM one-way ANOVA. **(D)** Phagocytic capacity of Mϕ1 and Mϕ2 was evaluated by flow-cytometry using fluorescent beads (experimental setup as in **B**). Macrophages were categorized into populations containing either 0, 1, 2, or 3+ beads. Bars depict mean ± standard deviation of 3 replicates. Data shown is 1 representative donor out of 4. Statistically significant differences compared to DMSO were tested using a one-way ANOVA with Dunnett's multiple test correction (*p < 0.05; **p < 0.01; ****p < 0.0001).

Next, we investigated whether HDAC inhibitors could divert monocytes from the classical Mϕ1 and Mϕ2 differentiation pathways to cell subsets exhibiting distinct characteristics including an increased bactericidal phenotype. Monocytes were exposed to low concentrations of HDAC inhibitors during our standard GM-CSF driven Mϕ1 or M-CSF driven Mϕ2 differentiation protocol (Figure 3A). Macrophages differentiated in the presence of HDAC inhibitors were more effective in restricting intracellular bacterial growth in both Mϕ1 and Mϕ2 compared to DMSO control treated cells. The pan-HDAC inhibitor TSA was slightly more effective in controlling intracellular *Mtb* infection than the selective class IIa HDAC inhibitors TMP195 and TMP269 (Figure 3B, red dots). Importantly, the observed reduction in *Mtb* outgrowth in macrophages differentiated in the presence of HDAC inhibitors was not due to decreased cell viability (Figure 3C, red dots) or a diminished capacity to phagocytose (Figure 3D), implying a strongly increased intrinsic capacity to control intracellular bacterial survival. Of note, TMP195 consistently increased the phagocytic capacity as well as the percentage of phagocytic cells, especially in Mϕ1 (Figure 3D and Figure S2), suggesting that the marginal reduction in bacterial load by Mϕ1 differentiated with TMP195 is considerably underestimating the increased bactericidal capacity induced by TMP195.

Since (1) upregulation of HDAC1 expression in *Mtb*-infected macrophages (Figure 1) has been postulated to involve the PKA-CREB-cJun signaling pathway (18) and (2) PKA inhibitor H-89 has previously been shown by us to counteract the manipulation of host signaling processes by *Mtb* (24, 66), we next investigated whether the restriction in bacterial outgrowth in macrophages differentiated in the presence of HDAC inhibitor could be further reduced by treating these macrophages subsequently with PKA inhibitors H-89 or 97i (an H-89 structural analog) following *Mtb*-infection in Mϕ2 (Figure 3B, gray dots). Because the effect of HDAC inhibitors on *Mtb* bacterial survival was more prominent in Mϕ2 than Mϕ1 (Figure 2B), we additionally investigated the putative additive effect of HDAC and PKA host-directed compound combination in Mϕ2 only. As shown in Figure 3B, a clear additive effect was observed between HDAC and PKA inhibitors in Mϕ2 with the strongest reduction in bacterial load in 97i-treated TSA-differentiated macrophages (median reduction of 69%), without resulting in significant toxicity (Figure 3C, gray dots).

In summary, these data propose a key role for chromatin remodeling by histone acetylation in orchestrating host defense in TB. Thus, functional inhibition of HDACs may be a promising (host-directed) therapeutic addition to drug-combination regimens already in use for TB.

### HDAC Inhibition Reduces Bacterial Burden *in vivo*

**Figure 4:**
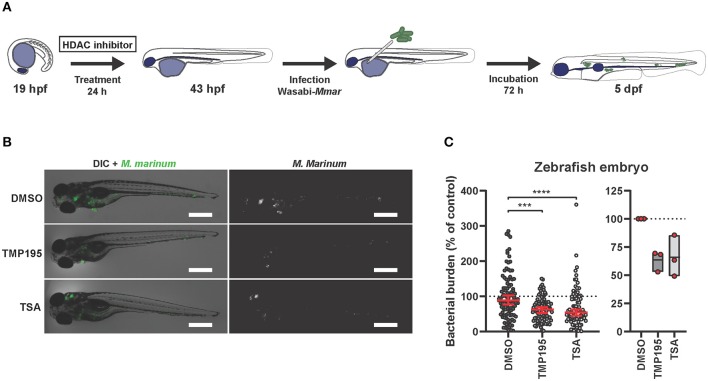
Zebrafish embryos exposed to HDAC inhibitors display reduced bacterial burden. **(A)** Outline of the experimental setup used in **(B,C)**. **(B)** Zebrafish embryos were at the 20 somite stage [19 h post fertilization (hpf)], exposed to TMP195 (10 μM), TSA (30 nM), or DMSO at equal v/v for 24 h. Embryos were subsequently infected by microinjection of 250–300 CFU *Mycobacterium marinum* (*Mmar*) expressing Wasabi fluorescent protein, into the Duct of Cuvier. Shown are representative infected larvae of each treatment group imaged for *Mmar* fluorescence at 3 days post infection. Scale bar indicates 500 μm. **(C)** Indicated is bacterial burden of all zebrafish larvae (left) or each independent experiment (right). Graphs represent zebrafish larvae from different parent couples used in 3 independent experiments, and treated with TMP195 (*n* = 92), TSA (*n* = 90), or DMSO at equal v/v (*n* = 102) expressed as a percentage of the DMSO control. Each dot indicates an embryo with horizontal line and whiskers in red representing median with 95% confidence intervals (left) or each dot indicates the mean of a single experiment with floating bars representing min. to max. while the horizontal line represents the mean of the 3 independent experiments (***p < 0.001; ****p < 0.0001).

To investigate the efficacy of HDAC inhibition *in vivo*, we employed a *Mycobacterium marinum* (*Mmar*) zebrafish embryo infection model. This model has been shown very effective for both fundamental and translational studies in the context of TB research (20–22, 67, 68). Since treatment with HDAC inhibitors during human macrophage differentiation followed by infection showed the highest drug efficacy as described above, we translated the human *in vitro* model to *in vivo* zebrafish embryos by treating them starting at the 20 somite stage, at which the first macrophages appear (69). At 24 h post treatment, embryos were infected with *Mmar* and 3 days after infection, zebrafish embryos were imaged to quantify bacterial burden (Figure 4A). Both TMP195 and TSA pre-treatment reduced bacterial burden *in vivo*, with an average reduction of 37 and 32%, respectively (Figures 4B,C). Importantly, no developmental toxicity was observed. These *in vivo* results strongly support and strengthen our *in vitro* human macrophage results (Figure 3B).

### HDAC Activity Regulates Cytokine Production by Macrophages in Response to *Mtb* Infection

Since HDAC activity has been implicated in guiding pro-vs. anti-inflammatory responses, we evaluated whether exposure to low concentrations HDAC inhibitors during monocyte differentiation altered the phenotype of pro-inflammatory Mϕ1 and anti-inflammatory Mϕ2. Expression levels of cell surface markers discriminating between Mϕ1 and Mϕ2 (CD14, CD1a, CD163, CD11b) or monitoring the activation status of macrophages (CD80 and CD86) were not affected, except for CD14 whose expression level was upregulated in a proportion of TSA-differentiated Mϕ1 (Figure S3A). Consistent with these findings, no morphological changes were observed in HDAC inhibitor-exposed Mϕ1 and Mϕ2 compared to DMSO controls (Figure S3B).

**Figure 5:**
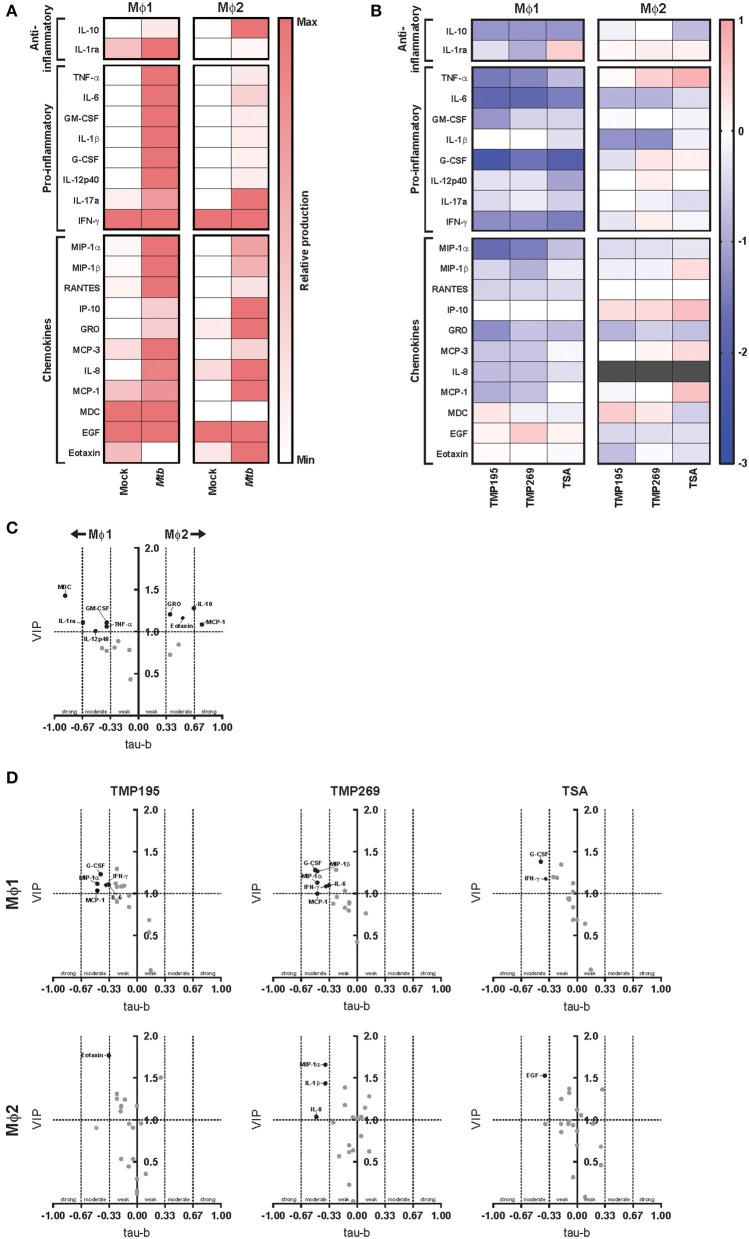
Exposure to low concentrations HDAC inhibitors during monocyte differentiation alters the cytokine/chemokine response of Mϕ1 and Mϕ2 upon *Mtb* infection. **(A)** Heat map displaying median cytokine/chemokine expression levels (of 4 different donors) in supernatants of standardly differentiated Mϕ1 and Mϕ2 24 h following *Mtb* or mock infection. Each row represents the relative expression of the indicated cytokine/chemokine using a white to red color scale. Of the 41 analytes measured, only cytokines/chemokines that changed in at least 3 out of 4 donors and exhibited a minimal median log_2_ fold change (FC) of 0.5 in a single comparison are shown. **(B)** Heat map displaying median log_2_ FC of cytokine/chemokine levels in supernatants of Mϕ1 and Mϕ2 of 4 different donors. In this experimental setup monocytes were exposed to TMP195 (300 nM), TMP269 (300 nM), TSA (30 nM), or DMSO at equal v/v during differentiation toward Mϕ1 and Mϕ2. Gray color depicts cytokine/chemokine levels that were detected above the linear range of the assay. **(C)** Experimental setup as in **(A)**. Variable Importance in Projection (VIP) scores of the first x-variate were extracted from each PLS-DA analysis and cytokine values ≥1 were considered relevant. In parallel, Kendall's tau-b correlation coefficients were calculated for each cytokine. Coefficients between 0–0.33, 0.33–0.67, and 0.67–1 were considered to have a weak, moderate and strong correlation, respectively. Every dot represents a cytokine/chemokine. Cytokines/chemokines with a VIP score >1 and demonstrating at least a moderate correlation are annotated in black. Annotated cytokines that were produced below or equal to a median concentration of 40 pg/ml are depicted by a diamond. **(D)** Experimental setup as in **(B)**. VIP scores and Kendall's tau-b correlation coefficients calculations as in **(C)**.

Before exploring whether exposure to low concentrations HDAC inhibitors during monocyte differentiation altered the cytokine/chemokine response of pro-inflammatory Mϕ1 and anti-inflammatory Mϕ2 upon *Mtb* infection, we first investigated the cytokine/chemokine responses of standardly differentiated Mϕ1 and Mϕ2 following infection with *Mtb*. Expression levels of 41 analytes were assessed in the supernatants of *Mtb*-infected Mϕ1 and Mϕ2 and compared to uninfected controls 24 h after infection. Both anti-inflammatory cytokines (IL-10, IL-1ra) and pro-inflammatory cytokines (TNF-α, IL-6, GM-CSF, IL-1β, G-CSF, IL-12p40, and IL-17a) were upregulated in Mϕ1 and Mϕ2 but, as expected, the induction of pro-inflammatory cytokines was superior in Mϕ1 compared to Mϕ2 whereas the induction of anti-inflammatory cytokine IL-10 was highest in Mϕ2, confirming and extending our previous findings (Figure 5A and Supplementary Table 1). To identify those cytokines/chemokines that highly discriminated between the innate responses of Mϕ1 and Mϕ2, a multilevel Partial Least Squares-Discriminant Analysis (PLS-DA) was performed. A PLS-DA rotates the PCA components to obtain maximal separation, producing Variable Importance in Projection (VIP) scores for each variable (e.g., analyte), reflecting the importance of each variable to the obtained separation (Figure S4A). In parallel, the association of each cytokine/chemokine secretion level with either Mϕ1 or Mϕ2 24 h after *Mtb* infection was calculated using Kendall's tau-b correlation test and plotted against the VIP scores (Figure 5C). These combined analyses identified MDC, IL-1ra, GM-CSF, TNF-α, and IL-12p40 as having moderate-to-strong correlations with *Mtb*-induced innate responses in Mϕ1, while for Mϕ2 MCP-1, IL-10, Eotaxin, and GRO were either uncovered or confirmed.

Next, we investigated the effect of exposure to low dose HDAC inhibitors during monocyte differentiation on the cytokine/chemokine response of Mϕ1 and Mϕ2 following *Mtb* infection (Figure 5B and Supplementary Table 1). Exposure to pan-HDAC inhibitor TSA and class IIa HDAC inhibitors TMP195 and TMP269 potently dampened the production of both anti- and pro-inflammatory cytokines as well as the majority of chemokines tested in Mϕ1 in response to *Mtb* infection. In contrast, exposure to HDAC inhibitors during differentiation had limited impact on the innate response of Mϕ2 with several cytokines/chemokines being slightly lowered in their production while the production of others was only marginally enhanced. To identify cytokines/chemokines most strongly associated with HDAC inhibition, PLS-DA analyses were performed separately for Mϕ1 and Mϕ2 and Kendall's correlation coefficients were calculated for every HDAC inhibitor-induced cytokine/chemokine response (Figure 5D and Figure S4B). In Mϕ1, G-CSF, and IFN-γ displayed a clear negative correlation with pan-HDAC and selective class IIa HDAC inhibition, while IL-6, MCP-1 and MIP-1α showed a negative correlation specifically with class IIa inhibition. In contrast, only weak correlations were observed for Mϕ2 (except for MIP-1α, IL-1β, and IL-8 upon TMP269 exposure), confirming a limited effect of exposure to low concentrations HDAC inhibitors during differentiation toward Mϕ2 on cytokines/chemokines responses following infection with *Mtb*.

To investigate whether RNA levels correlated with decreased cytokine and chemokine secretion, RNA levels encoding 8 molecules whose secretion was inhibited in response to HDAC inhibition (Figures 5B,D), were measured using qPCR in Mϕ1, since the most profound changes were observed in this macrophage subset (Figure S4C). For CCL2 (MCP-1) and CCL4 (MIP-1β), a clear correlation between transcript and chemokine secretion levels was found, in contrast to CXCL8 (IL-8) and CCL3 (MIP-1α). For several cytokines, such as IL-6, G-CSF, and IFN-γ, that were secreted in low amounts, we could not detect alterations in RNA levels. Interestingly, TNF transcripts could not be detected despite the fact that secretion of TNF-α levels was found to exceeded 1 μg/ml which suggests that post transcriptional regulation plays a major role in TNF secretion. Because both G-CSF and IFN-γ negatively correlated with HDAC inhibition in *Mtb*-infected Mϕ1 and IFN-γ is known to play a major role in TB pathogenesis (70), we further explored the possible role of IFN-γ in Mϕ1 that were differentiated in the presence of HDAC inhibitors (Figure S5A). Addition of IFN-γ to *Mtb*-infected Mϕ1 decreased the efficacy of HDAC inhibition (Figure S5B), without affecting cell viability (Figure S5C). Moreover, while HDAC inhibition during differentiation did not affect transcript levels of HDAC1 and HDAC 5 in *Mtb*-infected Mϕ1 (Figure S5D), presence of IFN-γ induced a significant downregulation of HDAC1 expression levels, particularly in Mϕ1 differentiated in the presence of TSA (Figure S5E). Together with its strong upregulation upon infection (Figure 1 and Figure S1), this supports an important role for HDAC1 during infection with *Mtb*.

Collectively, HDAC inhibition during macrophage differentiation profoundly downregulated inflammatory cytokine production induced by *Mtb* infection, particularly in Mϕ1. For several chemokines, this clearly correlated with lowered transcript levels while this correlation was absent for others, suggesting post-transcriptional modification also plays a role. Since *Mtb* can exploit host cytokine signaling networks for its survival and a delicate balance between pro- and anti-inflammatory cytokines is required to restrict *Mtb* proliferation (71), these data suggest that HDACs may affect the outcome of *Mtb* infection by altering infection-induced orchestrated cytokine/chemokine responses by innate immune cells.

## Discussion

Here, we report that histone deacetylase (HDAC) transcriptomic levels are strongly affected by *Mtb*-infection in primary human macrophages. Secondly, we report that broad chemical HDAC inhibition can enhance the antimicrobial response of both Mϕ1 and Mϕ2, while selective inhibition of class IIa HDACs prominently decreased bacterial outgrowth in Mϕ2. Thirdly, chemical inhibition of HDAC activity during differentiation polarized macrophages into a more bactericidal phenotype with a concomitant decrease in the secretion levels of inflammatory cytokines. Fourth, *in vivo* chemical inhibition of HDAC activity in *Mycobacterium marinum* infected zebrafish embryos, a well-characterized animal model for tuberculosis, significantly reduced mycobacterial burden *in vivo*, validating our *in vitro* findings in primary human macrophages. Collectively, these data identify HDACs as druggable host targets for HDT against intracellular *Mtb*.

Previous studies have suggested that *Mtb* can modulate host defense by epigenetic modifications to facilitate survival within the host cell (16, 18, 19). In this study, we observed that following *Mtb* infection, transcriptional levels of several HDACs representing different classes were differentially regulated in Mϕ1 but primarily in Mϕ2. Our findings, which are in agreement with (in this paper) independently analyzed results from Blischak et al. (64), identify HDAC enzymes as potential targets for immune modulation in infectious diseases. This idea is supported by similar expression levels of several HDACs when comparing Mϕ2 infected with a virulent *Mtb* strain to uninfected controls. Since macrophage differentiation states are known to be dynamic and flexible, macrophages represent highly interesting therapeutic targets, both in differentiated (e.g., Mϕ1 and Mϕ2) and in less differentiated stages. Interestingly, treatment with the pan-HDAC inhibitor TSA decreased bacterial survival in both *Mtb*-infected Mϕ1 and Mϕ2 while selective class IIa HDAC inhibitors TMP195 and TMP269 decreased *Mtb* survival predominantly in Mϕ2. This is one of the first studies comparatively analyzing HDT in Mϕ1 and Mϕ2 in a human infection model, indicating that downregulation of HDAC activity in the context of *Mtb* infection can be beneficial to host control of infection. The attenuated efficacy of class IIa HDAC inhibitors on *Mtb* survival in Mϕ1 might be explained by the lower basal expression levels of HDAC5 and HDAC7 in Mϕ1 compared to Mϕ2 (Figure S1A), which could suggest that the therapeutic window of these inhibitors is significantly larger in Mϕ2.

A recent cohort study in Uganda compared whole genome transcriptional profiles of *Mtb*-infected monocytes derived from peripheral blood of household contacts of TB patients who were resistant to *Mtb* infection (resisters) with individuals who were susceptible to *Mtb* infection [latent TB infection (LTBI)] (72, 73). Consistent with our observation that HDAC function is important for the innate immune response to *Mtb* infection, they showed that pathways controlled by HDACs were markedly differentially activated between the two study groups. The clinical potential of HDAC inhibition in the context of TB has already been proven by studies showing reduced bacterial burden in an *in vivo* mouse model using Tubustatin A, a HDAC6 inhibitor (74). Here, we significantly expand upon this work by demonstrating the potential of both a selective class IIa inhibitor and a pan-HDAC inhibitor, TMP195 and TSA, respectively, for treating mycobacterial infection in an *in vivo* model. Zebrafish embryos pre-treated with TMP195 or TSA, at concentrations not inducing developmental toxicity, showed a clear reduction in mycobacterial infection burden. A useful characteristic of the *Mmar* zebrafish embryo infection model is the lack of functional adaptive immune cells, thus allowing the assessment of innate immunity only (75). Despite the absence of T-cells, macrophage aggregates with granuloma-like features nevertheless are formed, a critical feature of TB (76). Therefore, our results support the effectivity of HDAC inhibitors during early stages of TB granuloma formation. Future work should be directed toward dissecting the effect of these HDT compounds in the presence of adaptive immunity and mature TB granulomas. Collectively, the independent data sets reported in our study and by others (74) strongly suggest that HDACs are an important factor in the innate immune response to *Mtb* infection, and that their inhibition can enhance antimicrobial activity of infected macrophages.

Interestingly, we found that targeting HDACs in monocytes during differentiation to either Mϕ1 or Mϕ2 strongly improved the ability of the host to control subsequent *Mtb* infection. In agreement with our finding, Guerriero et al., demonstrated in an *in vivo* mouse cancer model that treatment with class IIa HDAC inhibitor TMP195 increased the anti-tumor potential of macrophages by pharmacologic modulation of the macrophage phenotype (26). Importantly, in our *in vitro Mtb*-macrophage infection model, a combinatorial regimen of HDAC and PKA/PKB inhibitors resulted in a clearly additive effect in decreasing intracellular bacterial survival in Mϕ2 (Figure 3B). The PKA/PKB inhibitor H-89 has been shown to regulate a kinase network around AKT1/PKB-AS160-RAB14 that controls the intracellular survival of *Mtb* and *Salmonella* by manipulating phagosome maturation and actin remodeling (24, 66). Furthermore, PKA is known to be involved in numerous other signaling pathways associated with *Mtb* survival (77, 78). Moreover, PKA inhibition might also have impaired class IIa HDAC function by interfering with nucleocytoplasmic trafficking since PKA activation promotes nuclear import of HDAC4 by phosphorylation and inhibits class IIa HDAC nuclear export via the LKB1-SIK2/3 axis (79, 80). Demonstration of synergistic effects of TSA and PKA/PKB inhibitors is in line with results by Zhu et al. (81), who demonstrated upregulation of the PI3K-AKT1 signaling pathway in *Mtb*-infected THP-1 cells treated with TSA. Of note, Zhu et al. (81) reported TSA to promote *Mtb* survival but this discrepancy is likely explained by the use of the THP-1 cell line which requires stimulation with phorbol 12-myristate 13-acetate (PMA) as opposed to primary macrophages, as well as the higher concentration (625 nM) of TSA they used, which was highly toxic in our model. Our work is one of the first demonstrations of synergism (82) between different HDT compounds in the control of bacterial pathogens and provides an important avenue for further studies in this area. We speculate that the simultaneous targeting of mechanistically different host pathways underlies this synergism.

The kinetics and quantities of cytokines released by the host during infection is an important aspect influencing the outcome of immune responses against *Mtb* (71, 83). Surprisingly, while HDAC inhibition during monocyte differentiation restricted intracellular bacterial outgrowth more effectively in *Mtb*-infected Mϕ2 than Mϕ1 (Figure 3B), the cytokine/chemokine secretion profile was only moderately altered in Mϕ2 (Figure 4B). In contrast, Mϕ1 exposed during differentiation to HDAC inhibitors clearly displayed a less pro-inflammatory phenotype, raising the question which HDAC inhibitor-induced cytokine/chemokine profile is optimal for host resistance against *Mtb*. In line with this, the addition of IFN-γ, a protein known to be vital in TB pathogenesis (70), impaired the effect of HDAC inhibition on bacterial survival in Mϕ1. Lastly, TSA-enhanced Mϕ2 polarization was demonstrated to be dependent on TSA-induced autophagy (84), a process vital for the clearance of *Mtb* (85). Future work will need to explore the role of autophagy in *Mtb*-infected macrophages treated with HDAC inhibitors.

Interestingly, it has been shown that both silencing and chemical inhibition of class IIa HDACs induces the expression of transcription factor Nur77, an orphan nuclear receptor and immediate-early gene that regulates cellular proliferation, apoptosis, inflammation, and glucose metabolism (86). Nur77 has been demonstrated to promote anti-inflammatory function by rewiring the tricarboxylic acid (TCA) cycle in pro-inflammatory macrophages (87). Moreover, Nur77-deficiency was found to drive macrophage polarization toward a pro-inflammatory phenotype, characterized by increased IL-6, IL-12, and IFN-γ production, among others (88, 89). Despite the fact that specific effects of different HDACs on inflammatory profiles are just beginning to be elucidated (Table 1), we hypothesize that modulation of cytokine/chemokine secretion is a likely mechanism by which *Mtb* can evade from host defense and propose that this may be therapeutically counteracted by inhibiting HDAC-mediated transcriptional regulation (71).

Although, HDAC inhibitors are well-known for their regulation of transcriptional activity by histone deacetylation, their function may not be limited to modulating epigenetic changes. For example, it has been shown by Gregoire et al. that the function of transcription factor MEF2 can be inhibited through class IIa HDAC-mediated sumoylation (90, 91). Regulation of these alternative functions may also have contributed to an enhanced bactericidal capacity of HDAC inhibitor-treated macrophages. Therefore, a more complete understanding of the complex function of HDAC enzymes and their effect on cellular and immuno-modulatory processes will be necessary to understand the full therapeutic potential of their inhibitors.

In summary, our findings demonstrate that HDAC inhibitors offer the possibility to augment antimicrobial responses against *Mtb* infection. Moreover, they can act in synergy with other host-directed strategies and may well-synergize also with current antibiotics to improve TB treatment efficacy and to shortening TB therapies, a major goal in TB research. Although exploitation of HDACs as druggable targets for HDT against intracellular *Mtb* requires further work, our data clearly suggest that pharmacological targeting of host epigenetic regulation could be a promising strategy to improve the innate immune response against *Mtb*.

## Data Availability Statement

All datasets generated for this study are included in the article/Supplementary Material.

## Author Contributions

JM, BK, SV, MTH, and KW designed and performed the experiments and processed the experimental data. JM, BK, MTH, FV, TM, HS, AM, TO, and MCH contributed to the interpretation of the results. JM, MTH, TO, and MCH wrote the manuscript and designed the figures with input from FV. MCH supervised the project. All authors approved the final version of the manuscript.

### Conflict of Interest

The authors declare that the research was conducted in the absence of any commercial or financial relationships that could be construed as a potential conflict of interest.
