# Supplementary material for: Functional Inhibition of Host Histone Deacetylases (HDACs) Enhances in vitro and in vivo Anti-mycobacterial Activity in Human Macrophages and in Zebrafish
Source: Front Immunol. 2020 Feb 3;11:36. doi: 10.3389/fimmu.2020.00036 (PMC7008710; doi:10.3389/fimmu.2020.00036)
Supplement: Supplementary file 1 [file Image_1.pdf]

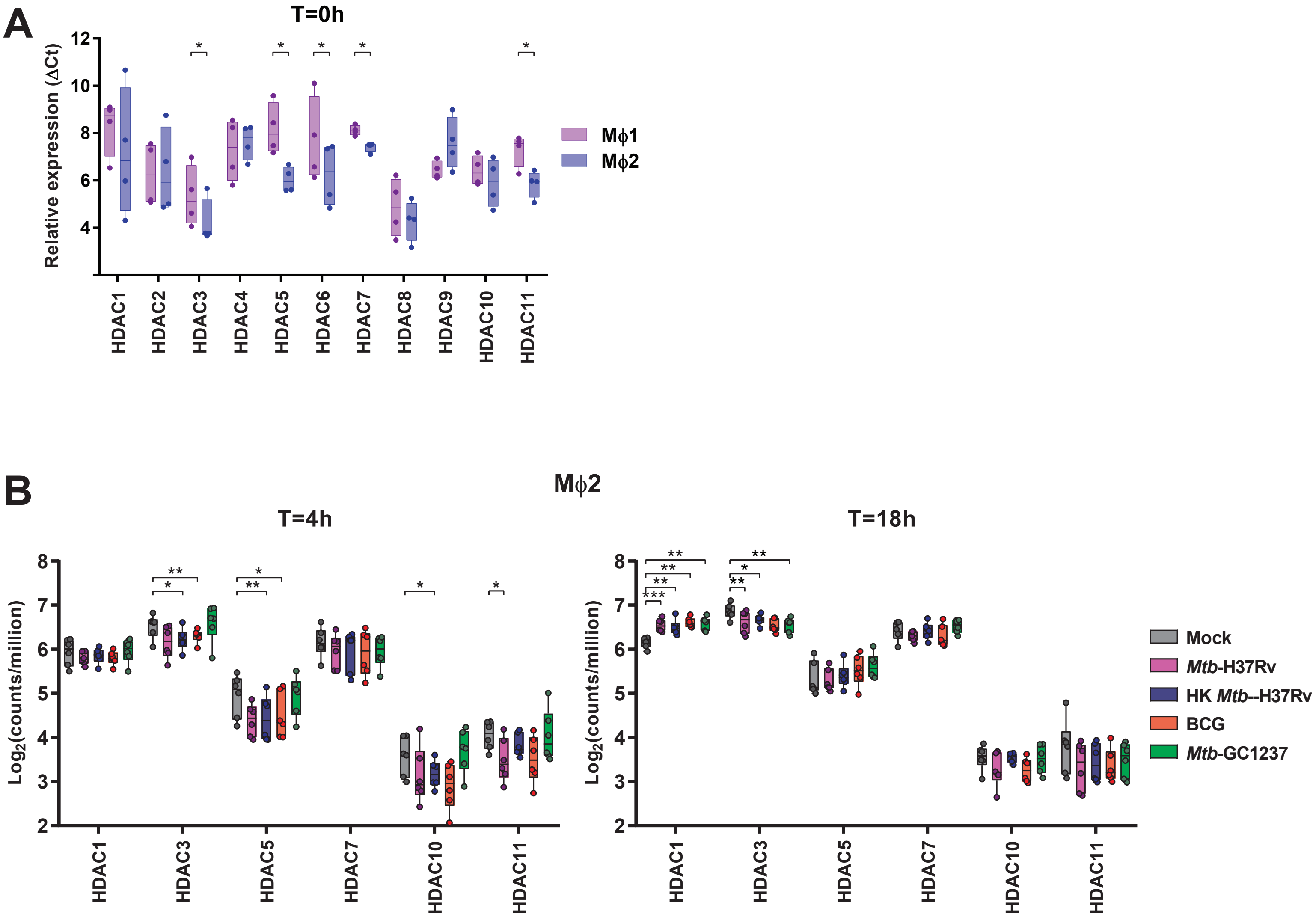

Figure S1. Validation of HDAC transcriptomic profiles in primary human macrophages following *Mtb* infection. A. Basal transcript levels of HDAC1-11 were determined in triplicate using qRT-PCR analysis at baseline (0h) in Mϕ1 and Mϕ2 derived from 4 different donors. Data was normalized to GAPDH and mean expression levels of triplicate samples were calculated for each donor. Box-and-whisker plots (min to max) show gene expression levels of the 4 donors where each dot represents a single donor. Significant differences between macrophage subsets were determined using a paired sample t-test. **B.** HDAC expression levels were extracted from a published RNA-sequencing dataset of infected Mϕ2 (Blischak et al., 2015). Mϕ2 of 6 different donors were mock infected or infected for 1h at MOI 2 with either *Mtb*-H37Rv, heat-killed *Mtb*-H37Rv, bacillus Calmette-Guérin (BCG) or *Mtb*-GC1237 and RNA was isolated 4 and 18h post-infection. Box-and-whisker plots (min to max) display the median log_2 counts per million of the 6 donors and each dot represents a single donor. Statistically significant differences compared to uninfected controls were tested using a repeated measure (RM) one-way ANOVA with Dunnett's multiple test correction. (* = $p<0.05$, ** = $p<0.01$ and *** = $p<0.001$).
