# Supplementary material for: Functional Inhibition of Host Histone Deacetylases (HDACs) Enhances in vitro and in vivo Anti-mycobacterial Activity in Human Macrophages and in Zebrafish
Source: Front Immunol. 2020 Feb 3;11:36. doi: 10.3389/fimmu.2020.00036 (PMC7008710; doi:10.3389/fimmu.2020.00036)
Supplement: Supplementary file 2 [file Image_2.pdf]

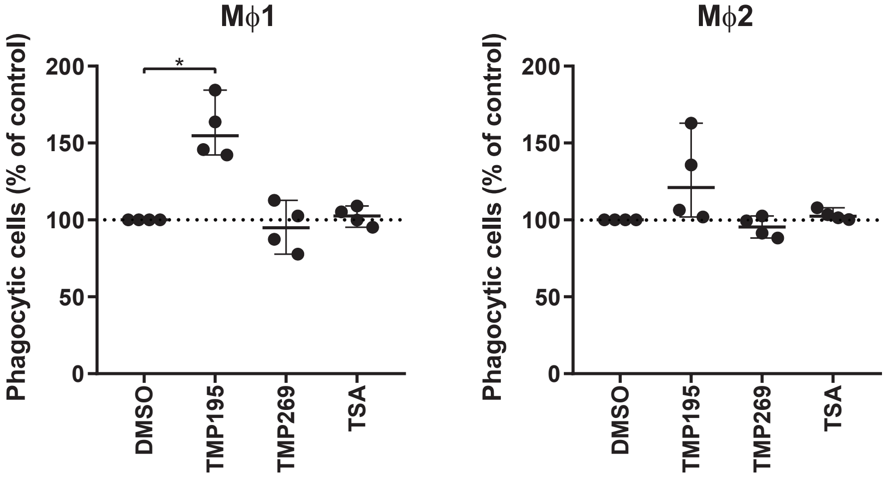


**Figure S2. A higher percentage of macrophages exhibit phagocytic capabilities when exposed during differentiation to low concentrations of HDAC inhibitor TMP195.** The percentage of Mϕ1 and Mϕ2 with phagocytic capabilities (containing 1+ beads) was evaluated by flow-cytometry using fluorescent beads (experimental setup as in Fig 3D). Dots represent the mean of 3 replicates of a single donor expressed as a percentage of the DMSO control. Horizontal lines indicate median percentage phagocytic cells of all 4 donors and whiskers represent 95% confidence intervals. Statistically significant differences compared to DMSO were tested using a RM one-way ANOVA with Dunnett's multiple test correction. (* = $p<0.05$).
