# Supplementary material for: Functional Inhibition of Host Histone Deacetylases (HDACs) Enhances in vitro and in vivo Anti-mycobacterial Activity in Human Macrophages and in Zebrafish
Source: Front Immunol. 2020 Feb 3;11:36. doi: 10.3389/fimmu.2020.00036 (PMC7008710; doi:10.3389/fimmu.2020.00036)
Supplement: Supplementary file 3 [file Image_3.pdf]

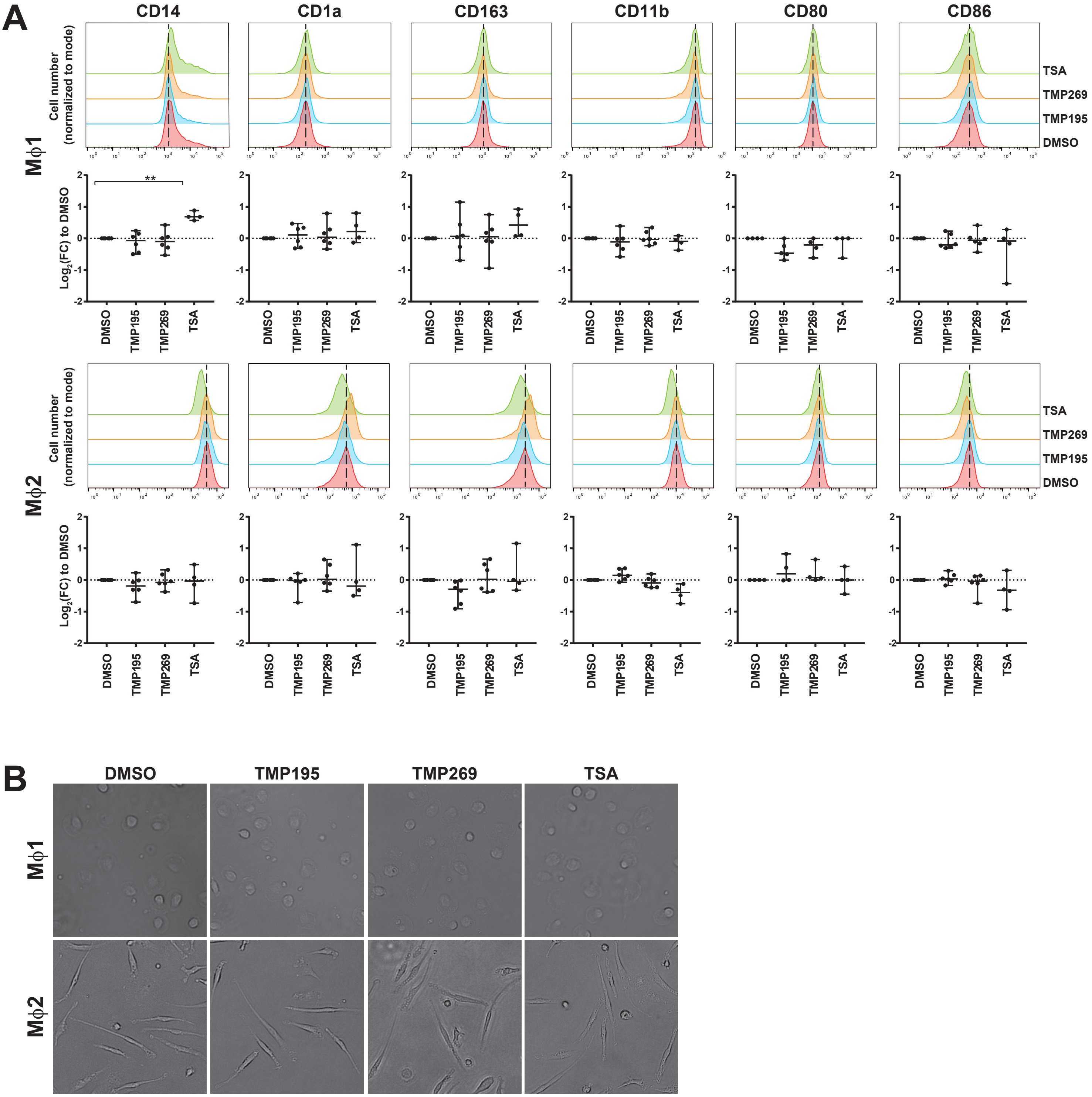

Figure S3. Expression of cell surface markers is unaltered on macrophages exposed to low concentrations of HDAC inhibitors during differentiation. Monocytes derived from 4-6 different donors were differentiated towards Mϕ1 and Mϕ2 while being exposed to TMP195 (300 nM), TMP269 (300 nM), TSA (30 nM) or DMSO at equal v/v for 6 days. **A.** Histograms depicting fluorescent intensities of cell surface markers (Top panel). For each cell surface marker the geometric mean fluorescent intensities (GMI) were calculated per donor. Dots represent the median $\log_2$ fold changes (FC) in response to chemical inhibition of HDAC activity during monocyte differentiation and is expressed as a percentage of the DMSO control. Horizontal lines indicate median fluorescent intensity values of all 4-6 donors and whiskers represent 95% confidence intervals. Statistically significant differences compared to DMSO were tested using a RM one-way ANOVA (** = $p<0.01$) (Lower panel). **B.** Bright field microscopy images showing the morphology of Mϕ1 and Mϕ2 exposed to TMP195 (300 nM), TMP269 (300 nM), TSA (30 nM) or equivalent volume of DMSO during differentiation (magnification 200x).
