# Supplementary material for: Functional Inhibition of Host Histone Deacetylases (HDACs) Enhances in vitro and in vivo Anti-mycobacterial Activity in Human Macrophages and in Zebrafish
Source: Front Immunol. 2020 Feb 3;11:36. doi: 10.3389/fimmu.2020.00036 (PMC7008710; doi:10.3389/fimmu.2020.00036)
Supplement: Supplementary file 4 [file Image_4.pdf]

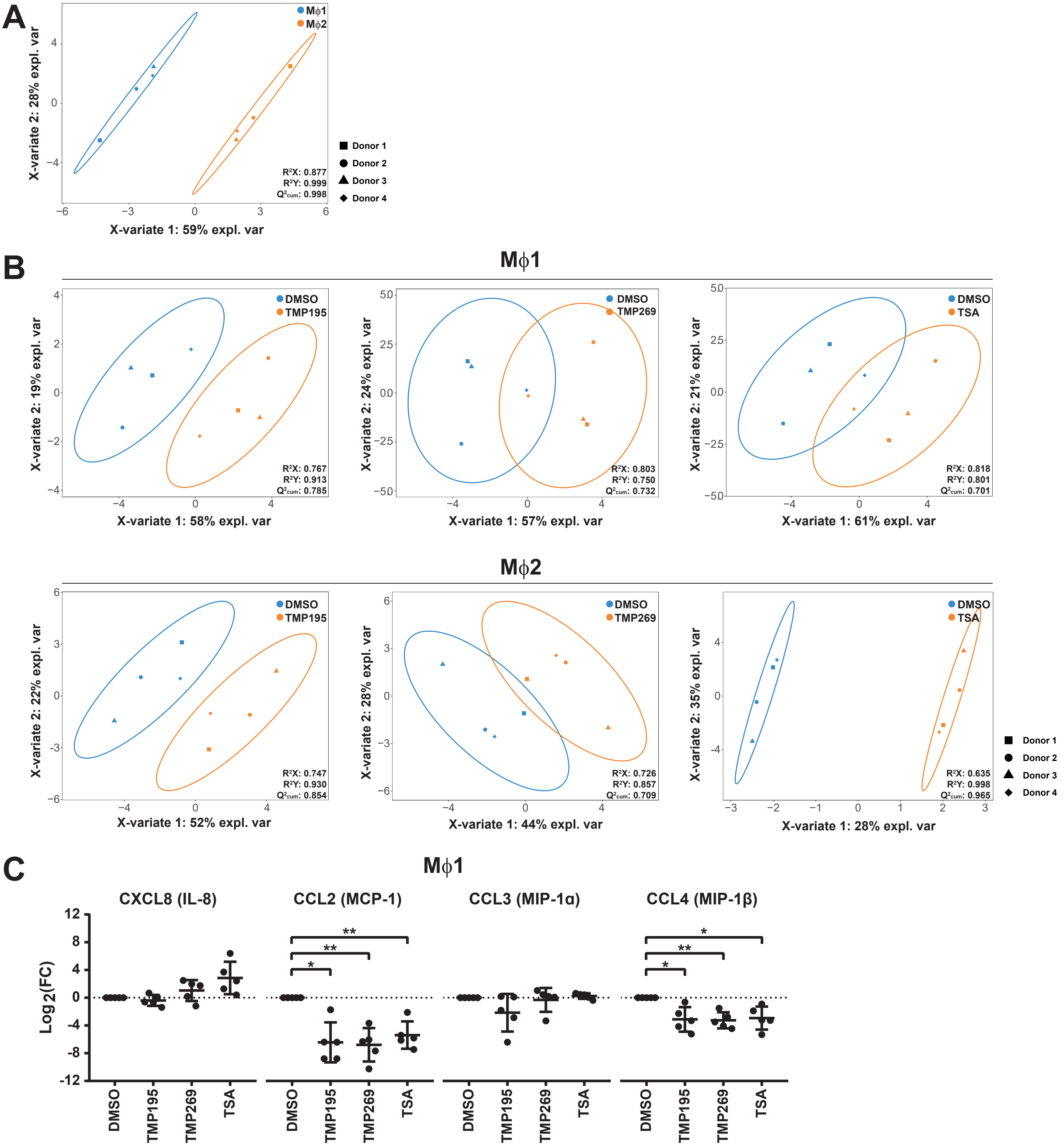

Figure S4. Inhibition of HDAC activity during monocyte differentiation modifies the cytokine/chemokine response of Mϕ1 and Mϕ2 upon Mtb infection. A. A multilevel PLS-DA model of cytokine profiles derived from standardly differentiated Mϕ1 and Mϕ2 24h following *Mtb* infection was generated using R package mixOmics. Model validity is indicated by the R2X, R2Y and Q2cum scores. **B.** Multilevel PLS-DA models of cytokine profiles derived from *Mtb*-infected Mϕ1 and Mϕ2 exposed to TMP195 (300 nM), TMP269 (300 nM), TSA (30 nM) or DMSO at equal v/v during differentiation were generated using R package mixOmics. Model validity is indicated by the R2X, R2Y and Q2cum scores. **C.** Transcript levels of CCL3 (MIP-1α), CCL4 (MIP-1β), CXCL8 (IL-8) and CCL2 (MCP-1) were determined in duplicate using qRT-PCR analysis in primary human *Mtb*-infected Mϕ1 derived from 5 different donors. Experimental setup as in B. Data was normalized to GAPDH (ΔCt) and mean expression levels of duplicate samples were calculated for each donor. Dot plots display $\log_2$ FC expression levels to their respective baseline controls (standardly differentiated Mϕ1), calculated using the formula 2-ΔΔCT. Each dot represents a single donor while horizontal lines indicate median $\log_2$ FC values of all 5 donors and whiskers represent 95% confidence intervals. Significant differences between treatments were determined using a RM one-way ANOVA with Dunnett's multiple test correction. (* = $p<0.05$ and ** = $p<0.01$).
