# Supplementary material for: Functional Inhibition of Host Histone Deacetylases (HDACs) Enhances in vitro and in vivo Anti-mycobacterial Activity in Human Macrophages and in Zebrafish
Source: Front Immunol. 2020 Feb 3;11:36. doi: 10.3389/fimmu.2020.00036 (PMC7008710; doi:10.3389/fimmu.2020.00036)
Supplement: Supplementary file 5 [file Image_5.pdf]

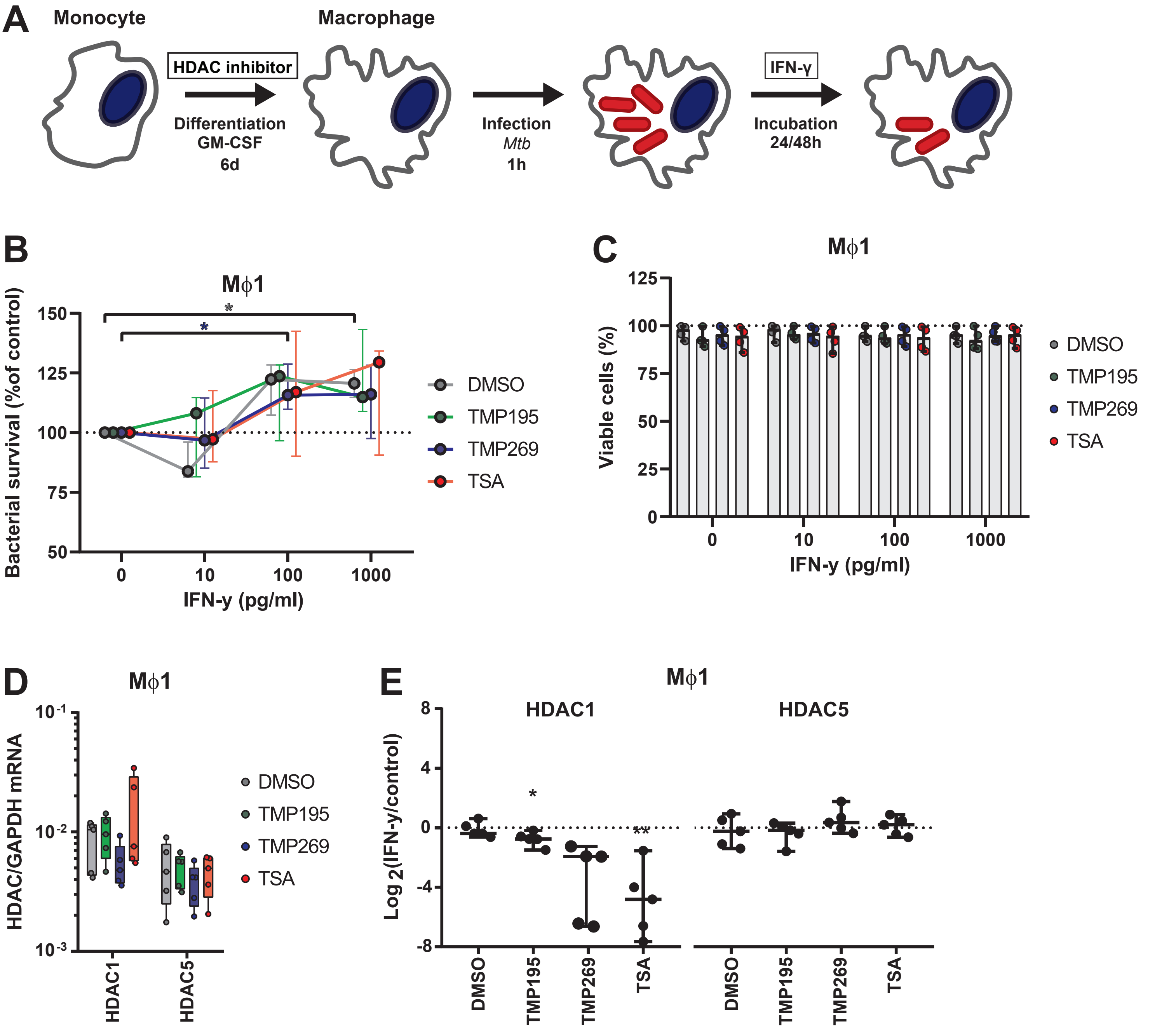

Figure S5. Inhibition of HDAC activity during monocyte differentiation modifies transcript levels of several cytokines/ chemokines in Mϕ1 upon Mtb infection. A. Outline of the experimental setup used in S5B-D. **B.** Monocytes derived from 4 different donors were differentiated towards Mϕ1 while being exposed to TMP195 (300 nM), TMP269 (300 nM), TSA (30 nM) or DMSO at equal v/v for 6 days. Differentiated Mϕ1 were subsequently infected with *Mtb* for 1h at MOI 10 and incubated for 48 hours with different amounts of IFN-γ. Dots depict the median bacterial survival of 4 donors expressed as a percentage of the DMSO control while whiskers represent 95% confidence intervals. Statistically significant differences were tested using a RM one-way ANOVA with repeated measures and Dunnett's multiple test correction. **C.** Cell viability measurement of *Mtb*-infected Mϕ1 (experimental setup as in A). Dots represent the mean of 2 viability assay replicates of a single donor expressed as a percentage of the DMSO control. Bars indicate median values of all 4 donors and whiskers represent 95% confidence intervals. Statistically significant differences were tested using a RM one-way ANOVA. **D.** Transcript levels of HDAC1 and HDAC5 were determined by qPCR in duplicate in *Mtb*-infected Mϕ1 from 5 different donors exposed to TMP195 (300 nM), TMP269 (300 nM), TSA (30 nM) or DMSO at equal v/v during differentiation. Data was normalized to GAPDH ((2-ΔCT(HDAC))/(2-ΔCT(GAPDH))) and mean expression levels of duplicate samples were calculated for each donor. Box-and-whisker plots (min. to max.) show gene expression levels of the 5 donors where each dot represents a single donor. Significant differences between treatments were determined using a RM one-way ANOVA with Dunnett's multiple test correction. **E.** Transcript levels of HDAC1 and HDAC5 were determined by qPCR in duplicate in *Mtb*-infected Mϕ1 from 5 different donors in the presence or absence of IFN-γ (1000 pg/ml). M 1 had been exposed to TMP195 (300 nM), TMP269 (300 nM), TSA (30 nM) or DMSO at equal v/v during differentiation. Data was normalized to GAPDH and mean expression levels of duplicate samples were calculated for each donor. Dot plots display $\log_2$ FC expression levels to their respective baseline controls (Mϕ1 in the absence of IFN-γ) calculated using the 2-ΔΔCT formula. Significant differences between presence and absence of IFN-γ were determined using a paired sample t-test (* = p<0.05, ** = p<0.01).
